# Cost-effectiveness and value of information analysis of NephroCheck and NGAL tests compared to standard care for the diagnosis of acute kidney injury

**DOI:** 10.1186/s12882-021-02610-9

**Published:** 2021-12-01

**Authors:** Elisabet Jacobsen, Simon Sawhney, Miriam Brazzelli, Lorna Aucott, Graham Scotland, Magaly Aceves-Martins, Clare Robertson, Mari Imamura, Amudha Poobalan, Paul Manson, Callum Kaye, Dwayne Boyers

**Affiliations:** 1 Health Economics Research Unit, University of Aberdeen, Aberdeen, UK; 2 Aberdeen Centre for Health Data Science, University of Aberdeen, Aberdeen, UK; 3 Health Services Research Unit, University of Aberdeen, Aberdeen, UK; 4 Institute of Applied Health Sciences, University of Aberdeen, Aberdeen, UK; 5 NHS Grampian, Aberdeen Royal Infirmary, Aberdeen, UK

**Keywords:** Acute kidney injury, Critical care, Cost-effectiveness, Diagnostic accuracy, Economic evaluation, Markov model, Nephrology

## Abstract

**Background:**

Early and accurate acute kidney injury (AKI) detection may improve patient outcomes and reduce health service costs. This study evaluates the diagnostic accuracy and cost-effectiveness of NephroCheck and NGAL (urine and plasma) biomarker tests used alongside standard care, compared with standard care to detect AKI in hospitalised UK adults.

**Methods:**

A 90-day decision tree and lifetime Markov cohort model predicted costs, quality adjusted life years (QALYs) and incremental cost-effectiveness ratios (ICERs) from a UK NHS perspective. Test accuracy was informed by a meta-analysis of diagnostic accuracy studies. Clinical trial and observational data informed the link between AKI and health outcomes, health state probabilities, costs and utilities. Value of information (VOI) analysis informed future research priorities.

**Results:**

Under base case assumptions, the biomarker tests were not cost-effective with ICERs of £105,965 (NephroCheck), £539,041 (NGAL urine BioPorto), £633,846 (NGAL plasma BioPorto) and £725,061 (NGAL urine ARCHITECT) per QALY gained compared to standard care. Results were uncertain, due to limited trial data, with probabilities of cost-effectiveness at £20,000 per QALY ranging from 0 to 99% and 0 to 56% for NephroCheck and NGAL tests respectively. The expected value of perfect information (EVPI) was £66 M, which demonstrated that additional research to resolve decision uncertainty is worthwhile.

**Conclusions:**

Current evidence is inadequate to support the cost-effectiveness of general use of biomarker tests. Future research evaluating the clinical and cost-effectiveness of test guided implementation of protective care bundles is necessary. Improving the evidence base around the impact of tests on AKI staging, and of AKI staging on clinical outcomes would have the greatest impact on reducing decision uncertainty.

**Supplementary Information:**

The online version contains supplementary material available at 10.1186/s12882-021-02610-9.

## Background

Acute kidney injury (AKI) incidence among the adult general population is estimated at about 150 per 10,000 per year [1]. Hospitalised patients are at greater risk following cardiac surgery (ranging from 8 to 40%), abdominal surgery (13.4%), and major trauma (21 to 24%) [2–5].

Early diagnosis and treatment can prevent AKI progression, which may reduce the risk of chronic kidney disease (CKD), and mortality [1, 6–8]. Patients who develop AKI in hospital are more likely to require renal replacement therapy (RRT) or intensive care unit (ICU) admission for kidney organ support and have longer length of stay (LOS). Cost implications to health services in England may be as high as £483 million per year [6].

In current standard care, AKI detection relies on monitoring changes in serum creatinine and urine output [9]. However, serum creatinine levels are not a precise indicator, and can take days to rise leading to delays in AKI recognition [2]. Novel biomarkers are intended to help detect AKI earlier, allowing initiation of prompt treatment with a care bundle to protect the kidneys, thereby improving outcomes and reducing healthcare costs. However, clinical and cost-effectiveness evidence for different biomarker tests is sparse, especially prior to admission to critical care [10–13]. This study uses a decision model to estimate the cost-effectiveness of four diagnostic biomarkers from a UK National Health Service (NHS) perspective. Value of information (VOI) analyses identify areas of greatest uncertainty where future research should be prioritised.

## Methods

### Patient population

The modelled population was UK hospitalised adults, at risk of AKI, who were having their kidney function monitored. The modelled population was designed to conform to the National Institute for Health and Care Excellence (NICE) assessment of diagnostic tests for AKI [14]. At model entry the cohort had a mean age of 63, and 54.3% were female, based on a published study of AKI incidence in a UK population [1].

### Interventions and comparators

Four biomarker tests were evaluated [14]. The NephroCheck test (Astute Medical) measures two biomarkers (tissue inhibitor of metalloproteinase 2 [TIMP-2] and insulin-like growth factor binding protein 7 [IGFBP-7]) in urine to calculate an AKI risk score. The threshold used for the NephroCheck test results to assess the risk of AKI was 0.3. The ARCHITECT urine NGAL assay (Abbott) is a chemiluminescent microparticle immunoassay to measure NGAL in human urine. The BioPorto NGAL test (BioPorto Diagnostics) is a particle-enhanced turbidimetric immunoassay to determine NGAL in either human urine or plasma (considered as two different tests). No restrictions were placed on the threshold for assessing the risk of AKI for the NGAL test results (see the Diagnostic accuracy section for more details). All tests were assessed in addition to standard clinical monitoring (e.g. serum creatinine and urine output monitoring), compared to standard clinical monitoring alone. The reference baseline levels of serum creatinine are defined according to current clinical criteria (Risk, Injury, Failure, Loss of kidney function, and End-stage kidney disease (RIFLE), Kidney disease: Improving Global Outcomes (KDIGO) and Acute Kidney Injury Network (AKIN)).

### Model structure

**Fig. 1:**
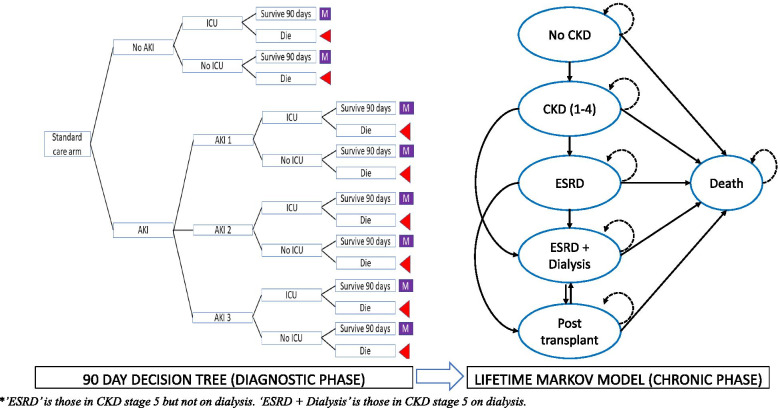
Model structure

A decision tree combined with a Markov cohort model was developed in TreeAge Pro (TreeAge Software, Williamstown, MA, 2019). The model structure (Fig. 1) was adapted from Hall et al. [10], who shared access to their model files under a ‘creative commons’ licence. The model structure was validated with clinical experts in nephrology and intensive care medicine.

#### Decision tree (up to 90 days)

The decision tree described the diagnostic accuracy and short-term health outcomes (admission to ICU, need for RRT, hospital LOS, and mortality) up to 90 days after test initiation. Tests may be true positive (TP), false negative (FN), true negative (TN) or false positive (FP). Biomarker tests may be beneficial if they can promptly and accurately identify AKI to enable appropriate early initiation of a protective care bundle which in turn can prevent further kidney damage. The benefits of prevention or a reduction in AKI severity (KDIGO stage) include reductions in LOS, need for RRT, ICU admission, development of CKD and the risk of mortality. All test positive patients are modelled to receive the care bundle but only those with a TP test result receive early treatment benefits. AKI prevention means keeping patients in the “No AKI” model pathway. It is assumed that FN tests incur the same risks as standard care, assuming that all positive AKI cases will eventually be identified using serum creatinine monitoring. Due to a lack of evidence, no negative effects of FP tests were assumed for the base case analysis but we recognise that identification and kidney support in AKI may not be risk free [15]. Scenario analyses explored an increased mortality risk associated with unnecessary removal of nephrotoxic treatments.

#### Markov model (lifetime horizon)

The Markov cohort model described CKD progression from early to subsequent end-stage renal disease (ESRD), need for dialysis, transplant, and mortality over a lifetime horizon from day 90 to death. The surviving cohort (at 90 days) enter the Markov model in either the “CKD (1-4)” or “No CKD” state. The cohort then transition between six health states: “No CKD”, “CKD (1-4)”, “ESRD”, “Post ESRD + dialysis”, “Post-transplant” and “Death” in annual model cycles, according to a set of transition probabilities. A half-cycle correction is applied. It was assumed that reversion to milder states (e.g. “No CKD”) was not possible. Those that had a failed transplant returned to dialysis, after which a subsequent transplant was possible. Other than the initial CKD risk, health state transitions are independent of 90-day AKI status. An annual mortality risk was modelled according to disease-specific [16–18] and age, and sex-adjusted general population risks [19].

### Model parameters

Full details of all model parameters (transition probabilities, relative risks, costs, and utilities) are provided in Additional file, Table 1.

#### Diagnostic accuracy

Test sensitivity and specificity were obtained from a systematic review and random-effects meta-analysis where possible. This model was chosen because of the heterogeneity across studies regarding different threshold values used for a positive NGAL test and variation in the classification systems used to determine AKI (KDIGO, RIFLE and AKIN). Table 1 describes the pooled diagnostic accuracy estimates obtained from 22 studies.

**Table 1:** Sensitivity and specificity data obtained from the systematic review

Test^**A**^	Parameter	Mean value (95% CI)	Mean (logit scale)	Standard error (logit scale)	Correlation for MVN distribution (logit scale)
NephroCheck (*N* = 7 studies)	Sensitivity	0.75 (0.58 to 0.87)	1.1178	0.3967	−0.824
	Specificity	0.61 (0.49 to 0.72)	0.4573	0.2567	
NGAL plasma (BioPorto) (*N* = 4 studies)	Sensitivity	0.76 (0.56 to 0.89)	1.1563	0.4615	−1.000
	Specificity	0.67 (0.40 to 0.86)	0.6863	0.5659	
NGAL urine Abbot ARCHITECT (*N* = 6 studies)	Sensitivity	0.67 (0.58 to 0.76)	0.7273	0.2047	−0.5168
	Specificity	0.72 (0.64 to 0.79)	0.9553	0.1909	
NGAL urine BioPorto (*N* = 8 studies)	Sensitivity	0.73 (0.65 to 0.80)	1.017	0.195	+ 0.526
	Specificity	0.83 (0.64 to 0.93)	1.562	0.511	

*MVN* Multi-Variable Normal

^A^Note that some studies evaluated more than one of the candidate tests

#### Clinical parameters

AKI incidence and severity were based on KDIGO staging (peak AKI status during hospitalisation), obtained from a 2012 cohort of people admitted acutely to hospital in the Grampian region of Scotland [1].

The impact of early delivery of a KDIGO care bundle on AKI was assumed to be that obtained from Meersch et al., a German trial of 276 NephroCheck positive patients, which reported a 16.6% (95% CI: 5.5 to 27.99%) absolute risk reduction in 72-h AKI for patients treated with a KDIGO care bundle compared to standard care [20]. The study also provided data to enable calculation of the impact of the care bundle on AKI severity (KDIGO staging). No comparable data were available for NGAL tests. Clinical expert advice indicated that NGAL may not detect kidney stress before damage occurs. It was therefore conservatively assumed that NGAL may reduce severity but could not prevent AKI.

The modelled hospital and post-discharge health outcomes associated with changes in AKI severity (need for ICU, 90 day mortality, hospital length of stay) were obtained from a re-analysis of published observational data from *N* = 17,630 patients admitted to Grampian (Scotland) hospitals in 2003, who were having their kidney function monitored through a blood test, and assumed to be at high risk of AKI [18, 21, 22]. Those with AKI, and those classified as having more severe AKI were more likely to need ICU care, had longer hospital LOS, and had a higher 90-day mortality risk. For those with AKI, the probability of developing CKD was calculated using hazard ratios from a recent systematic review and meta-analysis [23].

The modelled effect on outcomes of averting or reducing the severity of AKI through testing may be confounded with patient underlying characteristics. Therefore, based on clinical expert advice, and the published literature [20, 23, 24], we assumed that AKI mitigation/prevention leads to the full observed impact on CKD risk reduction obtained from the Grampian cohort data [23], some improvement (half the observed effect) in the need for ICU and hospital LOS and no improvement (none of the observed effect) in 90-day mortality [20, 24]. The extent to which observed associations are causal remains uncertain. Therefore, the proportion of the effect size applied in the model is varied in scenario analyses.

For the Markov cohort model, the proportion starting in the CKD state was calculated based on CKD prevalence (11.05% from the Grampian dataset) and hospital AKI severity. The remaining cohort with no initial CKD experienced an ongoing risk of developing CKD in the following cycles [10]. The remaining transition probabilities were obtained from a Swedish multi-centre cohort study, the SHARP trial, Scottish registry data and the UK Renal Registry [17, 25–27].

#### Costs

NHS perspective costs include the costs of test kits, staff time, hospital resource use up to day 90 (this included total number of days in hospital ward and ICU from the point of index admission up to day 90), hospital resource use after day 90 (including longer term hospital costs post discharge from hospital ward/ICU) and long term costs of CKD over a lifetime horizon.

Biomarker test costs included analysers, equipment, maintenance, consumables, staff time and training. Costs were based on manufacturer provided data, clinical expert opinion and unit costs for staff time (Additional file, Table 2). An additional three days of a preventative KDIGO care bundle was given to all test positive patients. This consisted of the avoidance of nephrotoxic agents, discontinuation of certain medications (ACE inhibitors and ARBs), regular monitoring of serum creatinine and urine output, steering clear of hyperglycaemia, avoiding radio contrast and intense hemodynamic monitoring. This was costed at £106.36, based on NICE guidelines for preventing AKI and included the costs of nephrologist and pharmacist time, intravenous fluids and clinical review of medications including those for blood pressure (ACE inhibitors, ARBs) [9]. Costs of LOS on a hospital ward, ICU, and RRT delivery were based on NHS reference costs [28].

Markov health states costs for those without CKD, included outpatient follow-up, as an average of those who had and had not received ICU care as part of their index admission [25]. The remaining health state costs were obtained from the SHARP trial, including outpatient, day-case and inpatient admissions [17]. Additional medication costs (immunosuppressant for a transplant patient, ESA for dialysis patients and blood pressure medications for dialysis patients) were obtained from the literature, NICE guidance and the BNF [16, 29–31]. All costs were incorporated in 2017/18 values, and inflated where necessary using the Cochrane and Campbell economic methods group online tool [32].

#### Quality adjusted life years (QALYs)

QALYs combine length (accounting for mortality) and quality of life into a single measure for use in decision-making. Utility data were obtained from an updated version of the systematic review published by Hall et al. [10]. For the acute decision tree phase, no new utility studies were identified. Therefore, utilities from Hall et al. were used. The review identified several health state utility values for the post discharge time period [33], CKD [34], ESRD [34] and dialysis [35] states. Where several utility studies were available, we prioritised those that used EQ-5D with a UK value set and larger sample sizes. All utilities used in the model were age and sex adjusted to allow quality of life to reduce naturally over time and to reconcile source study characteristics with the characteristics of the modelled cohort [36, 37].

#### Analysis

The decision model was analysed probabilistically using Monte Carlo simulation, with 1000 random draws. Costs and QALYs accruing beyond the first year were discounted at 3.5% per annum [38]. Incremental cost-effectiveness ratios (ICERs) were calculated for each test compared to the next best alternative, excluding those that were more costly and less effective than an alternative (dominated). Uncertainty was illustrated using cost-effectiveness acceptability curves (CEACs) and a comprehensive range of scenario analyses were carried out to explore the impact of key assumptions on the ICER. Subgroup analyses were conducted to explore the cost-effectiveness by parameterising the model using diagnostic accuracy data from several pre-defined subgroups (critical care only, post cardiac surgery only) (see Additional file, Table 5 and 6).

Expected value of perfect information (EVPI) and the expected value of perfect parameter information (EVPPI) were calculated for the model comparison of NephroCheck vs. standard care using the Sheffield Accelerated Value of Information (SAVI) tool [39, 40]. In this case, EVPI helps establish the economic value of future research (for example a new randomised trial) that could help inform the cost-effectiveness of NephroCheck vs. standard care by comparing the decision value under current and perfect information. Given a positive EVPI indicating that future research is worthwhile, we then used EVPPI analysis to identify research areas, specifically model parameters, where future research should be prioritised to have the greatest impact on reducing decision uncertainty [39]. To complete the EVPI and EVPPI calculations the target population size was assumed to be the number of AKI episodes in England in 2018 (564,738) [26], and the duration of time where the technology is relevant was assumed to be 10 years [39].

## Results

The base case analysis showed that none of the tests achieved an ICER <£20,000 per QALY gained compared to standard care (Table 2). Amongst the testing strategies, NephroCheck was the most promising test due to its potential ability to avert AKI.

**Table 2:** Base case cost-effectiveness results

	Cost	Incremental Cost	QALY	Incremental QALY	ICER (incremental)	ICER vs. standard care	p (C/E) @ 20 k	p (C/E) @ 20 k vs. standard care
Base case: Full associative effect of AKI mitigation on a) the risk of CKD within the first year, b) half the associative effect on the need for ICU, c) half the associative effect on hospital/ICU LOS, and d) no associative effect on 90-day mortality.								
Standard care (Scr)	£22,978	–	6.07277	–	–	–	64.5%	–
Test 1 (NephroCheck)	£23,016	£38	6.07313	0.00036	£105,965	£105,965	29.7%	32.0%
Test 3 (NGAL urine - BioPorto)	£23,049	Dominated	6.07290	Dominated	Dominated	£539,041	5.3%	11.0%
Test 2 (NGAL plasma - BioPorto)	£23,064	Dominated	6.07290	Dominated	Dominated	£633,846	0.3%	7.3%
Test 4 (NGAL urine - ARCHITECT)	£23,065	Dominated	6.07289	Dominated	Dominated	£725,061	0.0%	6.3%

Dominated: more costly and less effective; P(C/E): probability that a test is cost-effective at a threshold value of willingness to pay for a QALY of £20,000

Scenario analyses show that there is substantial residual uncertainty around the results. Full details of all scenario analyses are provided in the Additional file, Tables 3 and 4. Results are most sensitive to assumptions about the impact of AKI prevention on health outcomes. The extent to which preventing or reducing severity of AKI through early test guided protective KDIGO care bundles can truly reduce the risk of ICU admission, 90-day mortality, hospital length of stay and CKD risk is a major driver of model uncertainty. Assuming that the full observed relationship between AKI and these outcomes can be achieved through early detection and prevention (Scenario 1), NephroCheck is the most likely test to be cost-effective (98.5% probability if society is willing to pay £20,000 for a QALY gain). However, assuming none of the benefits in health outcomes can be achieved (Scenario 2), means none of the tests are cost-effective, demonstrating that it is not sufficient to prevent AKI, unless that prevention can definitively improve health outcomes.

Several scenario analyses increase the probability of NephroCheck being cost-effective (at a threshold value of £20,000 per QALY gained): including a daily excess AKI cost to patients in hospital ward/ICU over and above the hospital ward/ICU daily cost (Scenario 4, 98.8%), applying long-term costs and mortality risks that depend on whether the patient entered ICU or not (Scenario 6, 97.2%), assuming a lifetime excess CKD risk for AKI patients (Scenario 7, 55.5%), and applying a higher AKI prevalence (Scenario 10, 63.1%). On the other hand, some scenario analyses reduce the probability of the NephroCheck test being cost-effective: including removing RRT costs (Scenario 5, 27.7%), increasing the number of times a test is conducted to two (Scenario 11, 9%), and applying an additional mortality risk (RR = 1.5) to all FP test results (Scenario 12; 0%).

Given that our model base case assumes that NGAL cannot avert AKI, the probability of cost-effectiveness tends to be lower for the NGAL test strategies than NephroCheck across the scenario analyses. However, an analysis assuming that NGAL can also avert AKI, results in the BioPorto urine NGAL test being the optimal strategy (Scenario 3, 43.5%), though evidence to support the validity of this assumption is weak.

Subgroup analyses showed similar cost-effectiveness results for the critical care subgroup but low probabilities of cost-effectiveness in the cardiac care subgroup. Any suggestion of differences in cost-effectiveness across subgroups should be interpreted cautiously due to sparse diagnostic accuracy data in each subgroup, and due to a lack of subgroup specific data to inform downstream costs, utility and event probability parameters.

### Value of information analysis

**Fig. 2:**
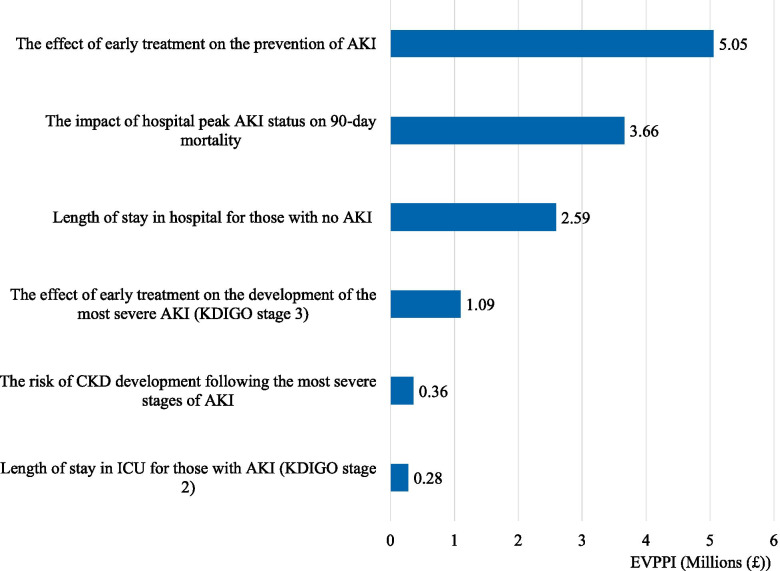
Prioritised areas for future research

The EVPI was £11.62 per patient and approximately £66 million at the population level over a ten-year time horizon, indicating that there is substantial value to be obtained from future research (such as well-designed randomised trials) to investigate the cost-effectiveness of NephroCheck vs. standard care (Fig. 2). Among individual model parameters, the EVPPI value was highest for the impact of early treatment on AKI prevention (£5.05 million) and the impact of AKI prevention on 90-day mortality (£3.66 million), indicating that research funding allocated to addressing these research questions would have the greatest impact on reducing decision uncertainty. The group population EVPPIs were £19.29 million for baseline probabilities, £8.98 million for costs and £0 for the utilities. This indicates there is adequate information available on utilities, but more research is required on overall AKI disease progression, downstream clinical events following AKI and its resource use implications.

## Discussion

### Interpretation of findings

This economic model compared the cost-effectiveness of four biomarkers NephroCheck, ARCHITECT urine NGAL and BioPorto urine and plasma NGAL to identify AKI in a UK hospital setting. The optimal, most cost-effective strategy was unclear, driven predominantly by the lack of clinical trial evidence of the impact of the biomarker tests on direct health outcomes such as ICU need, hospital LOS, development of CKD and mortality. Under base case assumptions, none of the biomarker tests were cost-effective. However, when exploring alternative analyses around uncertain parameters, the NephroCheck test had the greatest potential to achieve cost-effectiveness. That is because of NephroCheck’ s theoretical ability to pre-empt kidney damage and because the only evidence available to support an impact of a positive biomarker test guided implementation of an AKI care bundle is for NephroCheck [20].

The VOI analysis clearly shows that future research to resolve this decision uncertainty is worthwhile and that future research effort should be prioritised towards determining the clinical-effectiveness of novel biomarker tests in terms of AKI prevention through early intervention and 90-day mortality risks. Currently, clinical evidence points to some mitigation or aversion of AKI [20]. The observed data demonstrate that people with AKI (and more severe AKI) have poorer health outcomes than those without AKI (and with less severe AKI). However, it is not clear if averting AKI, or reducing its severity can fully remove the observed increased risk of adverse health outcomes associated with it. For example, Meersch et al. showed that early test guided application of a protective care bundle reduced AKI incidence and severity, but no beneficial effect on health outcomes was demonstrated. Similarly, an RCT of an early warning electronic alert system for AKI to trigger a renal consultation showed no evidence of a mortality benefit [24]. Hence, limited direct evidence exists to support an impact of biomarker tests on other health outcomes. Several trials are currently being conducted that are comparing NephroCheck guided initiation of a KDIGO care bundle to standard care (e.g. BigpAK1 trial, NEPHROCAR trial and a clinical trial in sepsis patients) [41]. These studies will report outcome data on AKI, 90-day mortality, need for RRT, ICU LOS, and hospital LOS. The data provided from these studies will be a valuable resource from which to inform future economic evaluations.

### Current literature

Hall et al. [10] also developed an economic model comparing different biomarker tests to assess the risk of AKI in ICU patients. The results for the biomarker tests in this study are less favourable to those reported in Hall et al. for several reasons. Our study scope focused on people not in ICU who could be considered for critical care rather than ICU patients where critical care is already being delivered. Therefore, the AKI prevalence is lower than in Hall et al. The higher the prevalence, the more likely the tests are to be cost-effective, as shown in our scenario analysis. This implies that careful consideration should be given to identifying subsets (e.g. post-major surgery) of those in hospital who would be most likely to benefit from testing and could be targeted in future trials. Furthermore, there were differences in test accuracy data between the reviews, with our review showing lower sensitivity for NephroCheck. This may be because new studies have become available since Hall et al. and the broader setting considered in our model. Finally, the approach to the estimation of costs is different; our analysis does not include an excess daily AKI cost for patients while in hospital, due to concerns about double counting costs already captured through AKI staging. An additional excess daily cost was added in scenario analysis. We also do not differentiate between the follow-up costs of ICU and non-ICU patients in the longer-term, though we explored this in scenario analyses.

### Strengths and limitations

A key strength is that the economic model is built on the basis of a comprehensive evidence synthesis to identify the diagnostic accuracy of the biomarker tests. The economic model was populated using both trial and real-world clinical registry data ensuring a high degree of external validity for the model inputs. The results are policy relevant and the VOI analysis in particular can help guide future research prioritisation and hence help avoid future research waste.

Our analysis was limited to the scope of the research question. For example, our results are only relevant to testing for AKI in a hospital setting. In addition, we recognise that NephroCheck and NGAL are two biomarkers among many. AKI biomarkers differ in what their measurement represents, across spectrums of inflammation, damage, and function loss at different time points. It may be that either a panel of biomarkers or sequential use of tests would be more clinically useful. However, it was not possible to evaluate these uses in our study. Additionally, given the broad heterogeneity of AKI causes, it is possible that further research will be able to delineate specific clinical circumstances or subsets of people in which biomarkers have the greatest potential to provide a cost-effective use of resources. Although our study had no ethnic restriction on inclusion criteria, future studies, especially clinical trials, should ensure diverse recruitment across ethnic and demographic populations. Furthermore, a separate analysis would be needed to assess the potential for stepping down care among those whose sole requirement for critical care is for kidney support.

This model did not account for CKD regression (i.e., a subset of people who have improving kidney function over time) [42] but used the conventional model for CKD where the majority are stable and a minority progress through CKD stages accumulating higher costs and worse outcomes, in line with previous kidney disease models [10]. It is unclear how this may or should influence mortality outcomes, or health care resource use. Furthermore, our model does not capture regression or progression between CKD stages (but applied average hospital care costs and utilities for this patient group) with a very small chance of progressing to CKD 5. Therefore, the impact of incorporating regression on cost-effectiveness findings is uncertain.

The results of the base case analyses should be interpreted cautiously because of the heterogeneous nature of the diagnostic accuracy studies in terms of NGAL threshold levels, timing of the sample collection, time of AKI diagnosis, definition of AKI, prevalence of AKI and definition of the population. Moreover, the clinical-effectiveness review identified no direct evidence about the incremental benefit of biomarker tests on clinically important and patient relevant health outcomes. We had to rely on a linked evidence approach, which contributed substantial decision uncertainty regarding cost-effectiveness.

## Conclusions

Current evidence is inadequate to support the cost-effectiveness of general use of biomarker tests. To minimise uncertainty about the cost-effectiveness of biomarker tests future research should be prioritised towards high quality randomised trials that target select patient subsets and assesses the added value of test guided use of AKI care bundles on clinically important and patient relevant health outcomes, in particular 90-day mortality.

## Supplementary Information

**Additional file 1: Supplementary Table 1**. Model parameters. **Supplementary Table 2**. Test costs. **Supplementary Table 3**. Scenario analyses description. **Supplementary Table 4**. Scenario analyses results. **Supplementary Table 5**. Sensitivity and specificity data obtained from the systematic review for the subgroup analysis. **Supplementary Table 6**. Results of the subgroup analyses.

## Data Availability

No new individual patient data were generated in support of this research. All cost-effectiveness and valuation of information model parameters are reported within the article and additional supplementary materials.

## Abbreviations

ACE: Angiotensin-converting enzyme
ARBs: Angiotensin receptor blockers
AKI: Acute kidney injury
AKIN: Acute Kidney Injury Network
BNF: British National Formulary
CEAC: Cost-effectiveness acceptability curve
CKD: Chronic kidney disease
EQ-5D: EuroQol five dimensional measure of quality of life
ESRD: End-stage renal disease
EVPI: Expected value of perfect information
EVPPI: Expected value of perfect parameter information
FN: False negative
FP: False positive
ICER: Incremental cost-effectiveness ratio
ICU: Intensive care unit
IGFBP-7: Insulin-like growth factor binding protein 7
KDIGO: Kidney Disease: Improving Global Outcomes
LOS: Length of stay
MVN: Multi-variable normal distribution
NHS: National Health Service
NICE: National Institute for Health and Care Excellence
QALY: Quality adjusted life year
RCT: Randomised controlled trial
RIFLE: Risk, Injury, Failure, Loss of kidney function, and End-stage kidney disease
RRT: Renal replacement therapy
SAVI: Sheffield accelerated value of information tool
SHARP: Study of Heart and Renal Protection
TIMP2: Tissue inhibitor of metalloproteinase 2
TN: True negative
TP: True positive
VOI: Value of information
