# Supplementary material for: Cost-effectiveness and value of information analysis of NephroCheck and NGAL tests compared to standard care for the diagnosis of acute kidney injury
Source: BMC Nephrol. 2021 Dec 1;22:399. doi: 10.1186/s12882-021-02610-9 (PMC8638090; doi:10.1186/s12882-021-02610-9)
Supplement: Supplementary file 1 — Additional file 1: Supplementary Table 1. Model parameters. Supplementary Table 2. Test costs. Supplementary Table 3. Scenario analyses description. Supplementary Table 4. Scenario analyses results. Supplementary Table 5. Sensitivity and specificity data obtained from the systematic review for the subgroup analysis. Supplementary Table 6. Results of the subgroup analyses. [file 12882_2021_2610_MOESM1_ESM.docx]

# Supplementary materials

## Supplementary Table 1 Model parameters

| **Parameter** | **Mean parameter value** | | **Standard Error** | **Distribution** | **Source** |
| --- | --- | --- | --- | --- | --- |
| **Starting age** | 63 | | **--** | Applied deterministically | Grampian data (1) |
| **Gender (female) %** | 54.3 | | **--** | Applied deterministically | Grampian data (1) |
| **Probability, length of stay and relative risks** | | | | | |
| **Incidence of AKI^A^** | | | | | |
| No AKI | 0.908 | |  | Remainder | Grampian data (2) |
| Any AKI | 0.092 | | -- | Beta (count),  (n = 4314, N=46884) | Grampian data (2) |
| AKI1  (given AKI) | 0.687 | | -- | Dirichlet | Grampian data (2) |
| AKI2  (given AKI) | 0.194 | | -- | Dirichlet | Grampian data (2) |
| AKI3  (given AKI) | 0.119 | | -- | Dirichlet | Grampian data (2) |
| **Probability of ICU admission** | | | | | |
| No AKI | 0.014 | | 0.0038 | Beta | Grampian data (1) |
| AKI 1 | 0.100 | | 0.0254 | Beta | Grampian data (1) |
| *RR of ICU admission vs. AKI1* | | | | | |
| AKI 2 | 1.423 | | 0.1082 | LN vs. AKI 1 | Grampian data (1) |
| AKI 3 | 1.930 | | 0.1096 | LN vs. AKI 1 | Grampian data (1) |
| **Probability of 90 Day mortality** | | | | | |
| No AKI | 0.049 | | 0.0069 | Beta | Grampian data (1) |
| AKI 1 | 0.215 | | 0.0347 | Beta | Grampian data (1) |
| *RR of 90-day mortality vs. AKI1* | | | | | |
| AKI 2 | 1.602 | | 0.0640 | LN vs. AKI 1 | Grampian data (1) |
| AKI 3 | 2.151 | | 0.0624 | LN vs. AKI 1 | Grampian data (1) |
| **Probability of requiring renal replacement therapy ^B^** | | | | | |
| No AKI, AKI 1 & 2 | 0 | | -- | -- | Assumption |
| AKI 3 | 0.552 | | -- | Beta (count)  (n=885; N=1603) | Truche et al. 2018 (3) |
| **Length of Stay (LOS) parameters** | | | | | |
| **Hospital LOS** | **Mean** | | **Median** |  |  |
| No AKI | 8.1 | | 3 | LN | Grampian data (1) |
| AKI 1 | 26.3 | | 14 | LN | Grampian data (1) |
| AKI 2 | 32.4 | | 18 | LN | Grampian data (1) |
| AKI 3 | 28.4 | | 17 | LN | Grampian data (1) |
| **ICU LOS^B^** |  | |  |  |  |
| No AKI | 2 | | 1 | LN | Bastin et al. 2013 (4) |
| AKI 1 | 4 | | 2 | LN | Bastin et al. 2013 (4) |
| AKI 2 | 8 | | 4 | LN | Bastin et al. 2013 (4) |
| AKI 3 | 26 | | 13 | LN | Bastin et al. 2013 (4) |
| **The effects of early adoption of a KDIGO care bundle (RR) ^C^** | | | | | |
|  | **Mean RR ^D^** | | **SE Log RR ^D^** |  |  |
| Any AKI | 0.768 | | 0.094 | LN | Meersch et al (5) |
| AKI 1  (given AKI) | 1.232 | | 0.180 | LN | Meersch et al (5) |
| AKI 2  (given AKI) | 0.868 | | 0.180 | LN | Meersch et al (5) |
| AKI 3  (given AKI) | 0.843 | | 0.356 | LN | Meersch et al (5) |
| Switch determining the proportional effect of AKI aversion | -- | | -- | Uniform. The uniform distribution takes a value between 0 and 1. | Due to the uncertainty around the extent of the effect of the RR (any AKI) from Meersch et al. the authors applied a proportional effect varying between 0 (no effect) and 1 (full effect). |
| Switch determining the effect of AKI mitigation | -- | | -- | Uniform. The uniform distribution either takes the value of 0 or 1. | Due to the uncertainty around whether AKI mitigation has an effect on final health outcomes, the authors applied a uniform distribution to the RR (AKI 1, 2, 3) that either took the value of 0 (no effect) and 1 (full effect). |
| **Parameters linking AKI and CKD** | | | | | |
|  | **Mean** | | **SE** |  |  |
| Prevalence of CKD (starting proportion) | 0.1105 | | -- | Beta (count);  n: 5,935  N: 53,691 | The starting proportion was dependent on underlying population CKD prevalence Grampian data (2) and the additional risk according to peak AKI severity from the initial hospitalization (see below hazard ratios obtained from See et al (6)). |
| Baseline incidence of CKD | 0.0044 | | 0.0003 | Beta | Rimes-Stigare et al (7) |
|  | **Mean HR** | | **Log SE** |  |  |
| Hazard Ratio of CKD given AKI1 | 2.32 | | 0.0363 | LN | See et al (6) |
| Hazard Ratio of CKD given AKI2 | 4.00 | | 0.5656 | LN |  |
| Hazard Ratio of CKD given AKI3 | 7.98 | | 0.9675 | LN |  |
| **Markov model transition probabilities** | | | | | |
|  | **Mean** | | **Standard Error** |  |  |
| Outpatient to CKD | 0.0044 | | 0.0003 | Beta | Rimes-Stigare et al (7) |
| CKD to death | 0.03 | | 0.002 | Beta | Kent et al (8) |
| CKD (survivors) to ESRD | 0.01 | | 0.001 | Beta |  |
| CKD (survivors) to ESRD + dialysis | 0.04 | | 0.002 | Beta |  |
| Remain with CKD |  | |  | Remainder |  |
| ESRD to death | 0.12 | | 0.005 | Beta | Kent et al (8) |
| ESRD (survivors) to ESRD + dialysis | 0.18 | | 0.006 | Beta |  |
| ESRD (survivors) to transplant | 0.09 | | 0.004 | Beta |  |
| Remain ESRD, no dialysis |  | |  | Remainder |  |
|  | **Alpha** | | **Beta** |  |  |
| Outpatient to death ^E^ | No ICU:  Year 1: 391  Years 2-5^F^: 748  ICU:  Year 1: 564  Year 2-5 ^F^: 964 | | No ICU:  Year 1: 4,824  Years 2-5^A^: 3,810  ICU:  Year 1: 4,651  Year 2-5: 3,322 | Beta (Count) | Lone et al (9)  Beyond 5 years, those in the “No CKD” (outpatient) state were assumed to have a mortality risk equal to that of the age and sex adjusted general population (ONS 2019 (10)) |
| ESRD + dialysis to Death | Year 1: 951  Year 3: 2116  Year 5: 2990 | | Year 1: 5178  Year 3: 3988  Year 5: 3254 | Beta (Count) | UK Renal Registry report (Table 1.17) (11) |
| ESRD + dialysis to Transplant | Year 1: 417  Year 3: 1056  Year 5: 1305 | | Year 1: 5712  Year 3: 5048  Year 5: 4939 | Beta (Count) |  |
| Remain in ESRD + dialysis |  | |  | Remainder |  |
| Transplant to ESRD + dialysis | Year 1: 4  Year 3: 16  Year 5: 26 | | Year 1: 487  Year 3: 475  Year 5: 431 | Beta (Count) | UK Renal Registry report (Table 1.17) (11) |
| Transplant to Death | Year 1: 8  Year 3: 16  Year 5: 31 | | Year 1: 483  Year 3: 475  Year 5: 426 | Beta (Count) |  |
| Transplant successful |  | |  | Remainder |  |
| **Costs** | | | | | |
| **Test costs** |  | |  |  |  |
| Astute medical  NephroCheck | £92.26 | | -- | Applied deterministically | See details on the cost calculations in Supplementary Table 2. |
| BioPorto (urine and plasma) | £59.55 | | -- | Applied deterministically |  |
| Abbott ARCHITECT | £66.87 | | -- | Applied deterministically |  |
| **Costs incurred up to 90 days (acute decision tree phase of the model)** |  | |  |  |  |
| 3 Days of KDIGO care bundle |  | |  |  |  |
| Intravenous fluids |  | |  |  |  |
| Intravenous sodium chloride 0.9% infusion 2litre bags (Terumo BCT Ltd). 1L per hour for 3 hours, thereafter 2L per day for 3 days (5 2 litre bags) | £22.14 | | -- | -- | Clinical expert opinion, BNF 2019 (12) |
| Band 6 nurse. Initial fluid: 10 minutes | £5.33 | | -- | -- | Clinical expert opinion, PSSRU 2018 (13) |
| Band 6 nurse. Fluid replacement: 5 minutes | £10.67 | | -- | -- | Clinical expert opinion, PSSRU 2018 (13) |
| Nephrologist review |  | | -- | -- |  |
| Hospital-based doctor, medical consultant. 30 minutes. | £54.00 | | -- | -- | Clinical expert opinion, PSSRU 2018 (13) |
| Pharmacist review |  | | -- | -- |  |
| Pharmacist, band 6 AfC. 20 minutes. | £15 | | -- | -- | Clinical expert opinion, PSSRU 2018 (13) |
| Stop blood pressure medication for 3 days | -£0.78 | | -- | -- | Clinical expert opinion, BNF 2019 (12). Based on the annual cost of blood pressure medication ^G^, and calculated over 3 days. |
| Total care bundle cost: | £106.36 | | £10.64 (mean x 10%) | Gamma |  |
| Hospital ward setting – daily cost | £313 | | £38.27 | Gamma | NHS reference costs 2017/18 (14) |
| ICU setting – daily cost | £1,395 | | £251.38 | Gamma | NHS reference costs 2017/18 (14). SD calculated using quartiles published from 2015/16 reference costs inflated to 2018 values. ^H^ |
| Excess daily cost of AKI^I^ | £298 | | £65.65 | Gamma | NHS reference costs 2017/18 (14). SD calculated using quartiles published from 2016/17 reference costs inflated to 2018 values  ^H^  (Applied in scenario analysis only) |
| Estimated daily cost of RRT^J^ | £197 | |  | Applied deterministically | NHS reference costs 2017/18 (14), assumes 48% on intermittent HD / 52% on continuous HD from ‘Adding insult to injury’ report 2009 (15). |
| **Follow up costs applied in the Markov model (day 90+)** |  | |  |  |  |
| *Annual follow up costs for the proportion of the cohort that were* ***not*** *admitted to ICU during the acute phase^K^* |  | |  |  |  |
| Year 1 | £3,954 | | £158 | Gamma | Lone et al. (9) |
| Year 2 | £2,864 | | £139 | Gamma |  |
| Year 3 | £2,547 | | £140 | Gamma |  |
| Year 4 | £2,277 | | £129 | Gamma |  |
| Year 5 | £2,090 | | £125 | Gamma |  |
| Year 6 | £1,794 | | £125 (assumption) | Gamma | Calculation based on Lone et al. (9) Costs from years 6 onwards applied in scenario analysis only |
| Year 7 | £1,618 | | £125 (assumption) | Gamma |  |
| Year 8 | £1,465 | | £125 (assumption) | Gamma |  |
| Year 9 | £1,331 | | £125 (assumption) | Gamma |  |
| Year 10 | £1,210 | | £125 (assumption) | Gamma |  |
| Year 11+ | £1,102 | | £125 (assumption) | Gamma |  |
| *Annual follow up costs for the proportion of the cohort that were admitted to ICU during the acute phase ^K^* |  | |  |  |  |
| Year 1 | £6,500 | | £198 | Gamma | Lone et al (9) |
| Year 2 | £4,183 | | £163 | Gamma |  |
| Year 3 | £3,975 | | £176 | Gamma |  |
| Year 4 | £3,774 | | £190 | Gamma |  |
| Year 5 | £3,315 | | £172 | Gamma |  |
| Year 6 | £2,806 | | £172 (assumption) | Gamma | Calculation based on Lone et al (9). Costs from years 6 onwards applied in scenario analysis only |
| Year 7 | £2,521 | | £172 (assumption) | Gamma |  |
| Year 8 | £2,274 | | £172 (assumption) | Gamma |  |
| Year 9 | £2,056 | | £172 (assumption) | Gamma |  |
| Year 10 | £1,861 | | £172 (assumption) | Gamma |  |
| Year 11+ | £1,685 | | £172 (assumption) | Gamma |  |
| **Health state specific costs applied in the Markov model** |  | |  |  |  |
| CKD 1-3 | £453 | | £33.53 | Gamma | Kent et al. 2015 (16) |
| CKD 4 | £441 | | £14.61 | Gamma | Kent et al. 2015 (16) |
| Weighted average (CKD 1-4) | £446 | | -- | -- | -- |
| ESRD (no dialysis) ^L^ | £590 | | £43.84 | Gamma | Kent et al. 2015 (16) |
| ESRD year 1 (with dialysis) | £21,328 | | £209.77 | Gamma | Kent et al. 2015 (16) |
| ESRD year 2 onwards (with dialysis) | £26,203 | | £54.45 | Gamma | Kent et al. 2015 (16) |
| Functioning transplant year 1 | £27,636 | | £329.84 | Gamma | Kent et al. 2015 (16) |
| Transplant follow up | £1,290 | | £97.43 | Gamma | Kent et al. 2015 (16) |
| *Additional medication costs applied to health states* |  | |  |  |  |
| ESRD year 1 (with dialysis) | £2,601^M^ | | -- | Applied Deterministically | NICE Guidance 2015 and BNF 2019 (12,17) |
| ESRD year 2 onwards (with dialysis) | £2,601^M^ | | -- | Applied Deterministically | NICE Guidance 2015 and BNF 2019 (12,17) |
| Functioning transplant year 1 | £10,623 | | -- | Applied Deterministically | NICE Guidance 2015 and BNF 2019 (12,17) |
| Transplant follow up | £9,063 | | -- | Applied Deterministically | NICE Guidance 2015 and BNF 2019 (12,17) |
| **Utilities** | | | | | |
|  | *Mean value from source* | *Age adjusted utility applied in the model* |  |  |  |
| **Utilities applied in the acute (Decision Tree) phase of the model** |  |  |  |  |  |
| ICU ^N^ | -0.402 | -0.402 | 0.02 | Normal | Kind et al. (Appendix B) (18) |
| Ward | 0.44 | 0.432 | 0.0259 | Beta | Hernández et al (16) |
| Discharge | 0.62 | 0.608 | 0.0268 | Beta | Hernández et al (16) |
| Acute dialysis decrement ^O^ | (0.11) | (0.11) | 0.02 | Beta | Wyld et al (19) |
| Death | 0 | 0 | -- | -- |  |
| **Utilities applied in the chronic (Markov) phase of the model** |  |  |  |  |  |
| Post discharge (year 1) | 0.666 | 0.655 | 0.016 | Beta | Cuthbertson et al. 2010 (20) |
| Post discharge (years 2-4) | 0.701 | 0.689 | 0.016 | Beta | Cuthbertson et al. 2010 (20) |
| Post discharge (year 5 onwards) | 0.677 | 0.665 | 0.017 | Beta | Cuthbertson et al. 2010 (20) |
| CKD (1-4) ^P, S^ | -- | 0.575 | -- | Beta | Nguyen et al. 2018 (21) |
| ESRD ^S^ | -- | 0.396 | -- | Beta | Nguyen et al. 2018 (21) |
| ESRD: HD^R^ | 0.560 | 0.551 | 0.033 | Beta | Liem et al. 2008;  Ara and Brazier, 2010 (22,23) |
| ESRD: PD^R^ | 0.580 | 0.564 | 0.043 | Beta | Liem et al. 2008;  Ara and Brazier, 2010 (22,23) |

**Abbreviations:** AKI: Acute Kidney Injury; BNF: British National Formulary; CKD: Chronic Kidney Disease; ESRD: End Stage Renal Disease; HD: Haemodialysis; HR: Hazard Ratio; ICU: Intensive Care Unit; LOS: Length of Stay; LN: Log Normal; NHS: National Health Service; NICE: National Institute for Health and Care Excellence; PD: Peritoneal Dialysis; RR: Relative Risk; RRT: Renal Replacement Therapy; SD: Standard Deviation; SE: Standard Error.

^A^ Note that incidence of AKI data are obtained from 2012 Grampian cohort, whereas probabilities of ICU admission and 90 day mortality are obtained from an earlier (2003) dataset.

^B^ For LOS in ICU, only median LOS information was available. For the purposes of parameterising the LN distribution and to account for the likely skewed nature of the data, it was assumed that the mean was twice the median. This ratio was obtained by dividing the mean LOS reported in Hall et al (24) by the median LOS for all AKI patients in the ICU setting.

^C^ Base case assumes that only NephroCheck guided care can avert AKI, not the NGAL tests.

^D^ Mean RR and SE log RR calculated by the authors using data from Meersch et al (5).

^E^ Average of ICU and hospitalised (non-ICU) mortality applied in the model base case analysis.

^F^ Converted to annual cycle specific probabilities for application in the model.

^G^ Blood pressure medication use is based on the study by Tan et a. 2016 and unit prices are obtained from BNF 2019. ACE inhibitor = £7.54 (=£35.74*0.211); ARBs = £6.70 (=£43.04*0.156); Calcium-channel blockers = £6.31 (=£28.80*0.219); Diuretics = 10.66 (=£21.92*0.487); Beta-blockers = £3.85 (=£15.52*0.248); Alpha-blockers = £1.35 (=£7.83*0.172). The average annual cost = £36.41.

^H^ Note that it has been necessary to obtain standard errors from older data as variability in costs are not reported in the 17/18 NHS reference costs; Standard errors calculated as SD / sqrt (N);

^I^ Applied in sensitivity analysis as an additional cost over and above the ward / ICU daily cost;

^J^ Assumed 3 sessions per week for intermittent HD and 1 session per day for continuous HD. Per day cost calculated as (cost per session x proportion on intermittent HD x 3 days per week) + (cost per session x proportion on continuous HD x daily) = (£271 x 0.48 x 3/7) + (£271 x 0.52 x 1) = £196.67 per day on average.

^K^ Note that the base case analysis applies the average of ICU and hospital, with differential costs applied as a sensitivity analysis to the proportion who require ICU care and hospital care in the initial 90-day phase

^L^ ESRD reported as CKD stage 5 in Kent et al.(8)

^M^ Cost of medications in the ESRD (with dialysis) health state was based on the total annual cost of both ESA medication and blood pressure medication. ESA medication use was based on UK renal registry report 2019 (11) (proportion of HD (87.5%) and PD (12.5%) on ESA (HD: 92.6%; PD: 78.6%) and median dose (international units (IU)) per week (HD: 8000; PD: 4000)), and unit prices obtained from BNF 2019 (average price per IU for NeoRecormon (£0.007) and Aranesp (£0.007) (12)).

^N^ Assumed standard error equal to 5% of the mean utility for an unconscious patient.

^O^ Decrement applied to utility in ward only.

^P^ A weighted average utility value (with proportions based on Nguyen et al. 2018 (21)) across the CKD stages 1-4.

^R^ For application in the model, the ESRD (dialysis) utility is applied as the weighted average utility based on the proportion of long-term dialysis delivered as HD and PD, obtained from the UK renal registry report, 2018 (25).

^S^ The study reports utility decrements only and the mean utility applied in the model is back calculated using the utility decrement from Nguyen et al. (21) applied to age and sex-adjusted UK general population norms.

##

## Supplementary Table 2 Test costs

|  | **Astute medical**  **NephroCheck** | **BioPorto (urine and plasma)** | **Abbott ARCHITECT** |
| --- | --- | --- | --- |
| **Platform (Astute 140 Meter), NephroCheck only because expert opinion indicated that the required analyser is not routinely available in UK hospitals** | | | |
| Cost | £3000 | -- | -- |
| Expected service life | 5 | -- | -- |
| Equivalent annual cost (EAC) | £664.44^E^ | -- | -- |
| **Subtotal: Platform (cost per test)** | **£0.53^A^** | **--** | **--** |
| **Subtotal: Equipment (cost per test)** | **£49.80^B^** | **£20.00^C^** | **£25.71^D^** |
| **Subtotal: Maintenance/consumables (cost per test)^F^** | **£4.23** | **£1.90** | **£3.51** |
| **Staff resource use** | | | |
| Time to conduct test (sample preparation + time to get result) (minutes) | 20 | 20 | 20 |
| Time to interpret test (minutes) | 5 | 5 | 5 |
| Prepare urine sample: nurse band 5 (minutes) | 15 | 15 | 15 |
| Bring urine sample to laboratory: porter (minutes) | 15 | 15 | 15 |
| Staff time for testing (per test) | £14.67 | £14.67 | £14.67 |
| Staff for interpreting (per test) | £6.89 | £6.89 | £6.89 |
| Staff to prepare urine sample (per test) | £9.25 | £9.25 | £9.25 |
| Delivery to lab (per test) | £6.82 | £6.82 | £6.82 |
| **Subtotal: staff costs (per test)** | **£37.62** | **£37.62** | **£37.62** |
| **Staff training ^G^** | | | |
| Assumed average turnover (years) | 5 | 5 | 5 |
| Time for training (minutes) | 90 | 30 | 30 |
| Total training costs | £438.00 | £146.00 | £146.00 |
| EAC of total training | £97.01 | £32.34 | £32.34 |
| **EAC of total training per test** | **£0.08** | **£0.03** | **£0.03** |
| **TOTAL COST** | **£92.26** | **£59.55** | **£66.87** |

^A^ Assuming the number of tests performed annually is 1253 (Hall et al. 2018 (24)), based on throughput at the ICU department of St James teaching hospital, Leeds. This is likely a conservative estimate of throughput that might be observed outside the ICU department and likely reflect the maximum bound of the allocated platform cost per test.

^B^ NephroCheck single use test cartridge.

^C^ BioPorto NGAL test.

^D^ ARCHITECT Urine NGAL Test Reagent 100-test kit (produces 80 tests) (Source: Company submitted request for information to NICE)

^E^ £644.44=£3000/((1-(1.035)^-5)/0.035), where 3.5% is the discount rate applied to the platform cost.

^F^ Sourced from manufacturers.

NephroCheck: £4.23 (=£2.50 (paper roll) / 25 (number of tests in kit) + £100 (Liquid quality control (one per kit)) / 25 (number of tests in kit) + £80*2 (Electronic quality control (every 6 months) / number of tests performed per year in hospital laboratory (=1253, in St. James's University Hospital, Leeds, source: Hall et al. 2018 (24))).

BioPorto: £1.90 (=£385 (NGAL Calibrator) / 300 (number of tests in kit) + £185 (NGAL Control kit) / 300 (number of tests in kit)).

Abbott/Alinity: £3.51 (=£165 (ARCHITECT Urine Calibrator kit) / 80 (number of tests a kit can produce) + £115 (ARCHITECT Urine Control kit Reaction vessels) / 80 (number of tests a kit can produce) + £0.01 (bulk solutions)).

^G^ Staff training time for all tests was based on information provided by the manufacturers where possible. For NephroCheck, training takes 1-2 hours, therefore, we assumed that on average training would take 1.5 hours (NICE’s request for information document). Training was assumed 30 minutes for all NGAL tests because the manufactures stated that only “limited training” (BioPorto) or time “to read the instructions for use” (Abbott) would be required. The total training cost was based on the total cost of training staff that would be conducting and interpreting the test results.

## Supplementary Table 3 Scenario analyses description

| **Parameter / assumptions** | **Base case Value** | **Main analysis justification / source** | **Sensitivity / scenario analyses** | **Scenario analysis reference** |
| --- | --- | --- | --- | --- |
| **Scenario analyses applied to base case analysis** | | | | |
| Proportion of the RR of ICU admission (AKI vs. none) that can be achieved by averting AKI | 0.5 | Based on clinical expert opinion | Varied between 0 and 1 | Scenario 1: Averting or reducing severity of AKI leads to **full** improvement in health outcomes  Scenario 2: Averting or reducing severity of AKI leads to **no** improvement in health outcomes |
| Proportion of the HR of CKD (AKI vs. none) that can be achieved by averting AKI | 1 | Based on clinical expert opinion / See et al (6) | Varied between 0 and 1 |  |
| Proportion of the RR of 90-day mortality (AKI vs. none) that can be achieved by averting AKI | 0 | Based on Meersch et al, who show effects on AKI, but not on mortality. Similar data from Wilson et al (5,26) | Varied between 0 and 1 |  |
| Proportion of the difference in hospital and ICU length of stay (AKI vs. none) that can be achieved by averting AKI | 0.5 | Based on clinical expert opinion | Varied between 0 and 1 |  |
| Impact of AKI stage on hospital and ICU length of stay | Duration applied by AKI stage | Based on observational data from Grampian (1) | Duration assumed not to vary by stage, with same durations applied to all AKI stages based on average from Grampian observational data (1) |  |
| Impact of AKI stage on the probability of ICU admission | Probability applied by AKI stage | Based on observational data from Grampian (1) | Probability assumed not to vary by stage, with same probability applied to all AKI stages based on average from Grampian observational data (1) |  |
| Impact of AKI stage on the probability of developing CKD | HR applied by AKI stage | Based on systematic review and meta-analysis from See et al (6) | HR assumed not to vary by stage, with same HR applied to all AKI stages based on Sawhney 2017 (1) |  |
| Impact of AKI stage on the probability of 90-day mortality | Average probability applied for all AKI stages | Based on a lack of evidence that changing AKI severity can impact directly on mortality, as per Meersch et al. (5) | Probabilities applied by AKI stage to explore uncertainty in this assumption |  |
| Potential for NephroCheck biomarker test to avert AKI (vs. standard care) | RR AKI = 0.77 | Based on Meersch et al. (5) | Also applied to NGAL tests | Scenario 3: NGAL and NephroCheck can both avert AKI |
| AKI excess cost per day in hospital / ICU | No excess cost applied | Conservative approach to ensure avoidance of double counting | Additional hospital excess bed day cost applied as per Hall et al. to all patients (ICU / ward) (24) | Scenario 4: as per Scenario 3 (full associative effect) with additional AKI costs. |
| Additional costs associated per day on RRT | Yes | Based on HRG costs (14) | No additional costs of RRT | Scenario 5 |
| Impact of AKI on long term follow up costs beyond 90 days | None (ratio =1) | Conservative assumption | All long-term Markov model costs multiplied by 1.15 as per Hall et al (24) | Scenario 6  Differential long term outpatient cost and mortality applied according to whether patient entered ICU or not. |
| Long term outpatient follow-up costs, up to 5 years | Average of hospitalised and ICU patients | Based on average of two cohorts from Lone et al. (9) | Differential cost streams applied for 5 years according to whether cohort admitted to ICU in first 90 days, based on Lone et al. (9) |  |
| Long term outpatient follow-up costs, after 5 years | No additional costs applied | Assumption that patients surviving post ICU to 5 years will incur no further excess costs | Additional annual costs applied for full life-time based on extrapolation of Lone et al. data, applied separately to those who had ICU / no ICU admission at index hospitalisation. |  |
| Impact of ICU admission on long-term mortality | Average of hospitalised and ICU patients | Lone et al. | Differential mortality applied according to whether cohort admitted to ICU |  |
| Duration by which AKI event can impact on excess CKD risk | 90 days + 1 year | Assumption | Assume additional risk of CKD development over full life-time horizon | Scenario 7 |
| Discount rate (Cost) | 3.5% | NICE guidelines | Varied 0% - 6% | Scenario 8 (0%)  Scenario 9 (6%) |
| Discount rate (QALY) | 3.5% | NICE guidelines | Varied 0% - 6% |  |
| Source of AKI prevalence data | 9.2% | Grampian data for hospitalised patients at risk of AKI | Alternative source: obtained directly from systematic review studies | Scenario 10 |
| Number of times test is used | 1 | Based on NICE scope (27) | All tests conducted twice | Scenario 11 |
| RR of 90-day mortality for FP test results | 1 | Assumes no additional risk of unnecessary fluid resuscitation | Apply an additional RR=1.5 to explore impact on results. | Scenario 12 |
| Test capital and training costs in test cost | Included | As per company advice | Exclude in sensitivity analysis, assuming all capital equipment required is available for all tests (including NephroCheck) | Scenario 13 |
| Source of ICU utility data | -0.402 | Kind et al (unconscious patient) (18) | Average of unconscious  (-0.402) and utility at discharge from ICU reported in the Practical trial (Hernandez et al) (16) | Scenario 14 |
| Long term outpatient utility | Varies by year | Long term utility implication of hospitalisation / ICU, based on Hall et al. (24) | General population norms, assuming quicker recovery. | Scenario 15 |

## Supplementary Table 4 Scenario analyses results

| **Scenario** | **Cost** | **Incremental cost** | **QALY** | **Incremental QALY** | **ICER (incremental)** | **ICER vs. standard care** | **p (C/E) @ 20k** | **p (C/E) @ 20k vs. standard care** |
| --- | --- | --- | --- | --- | --- | --- | --- | --- |
| **Scenario 1: Full associative effect of AKI mitigation on health outcomes** | | | | | | | | |
| Standard care (Scr) | £23,114 | -- | 6.08592 | -- | -- | -- | 0.7% | -- |
| Test 3 (NGAL urine - BioPorto) | £23,199 | Ext Dom | 6.09125 | Ext Dom | Ext Dom | £15,974 | 0.5% | 55.8% |
| Test 2 (NGAL plasma - BioPorto) | £23,214 | Ext Dom | 6.09137 | Ext Dom | Ext Dom | £18,364 | 0.3% | 50.3% |
| Test 4 (NGAL urine - ARCHITECT) | £23,215 | Dominated | 6.09080 | Dominated | Dominated | £20,721 | 0.0% | 46.0% |
| Test 1 (NephroCheck) | £23,223 | £109 | 6.11360 | 0.02768 | £3,941 | £3,941 | 98.5% | 99.1% |
| **Scenario 2: No associative effect of AKI mitigation on health outcomes** | | | | | | | | |
| Standard care (Scr) | £23,012 | -- | 6.07534 | -- | -- | -- | 100.0% | -- |
| Test 3 (NGAL urine - BioPorto) | £23,094 | £82 | 6.07534 | Dominated | Dominated | Dominated | 0.0% | 0.0% |
| Test 2 (NGAL plasma - BioPorto) | £23,110 | £16 | 6.07534 | Dominated | Dominated | Dominated | 0.0% | 0.0% |
| Test 4 (NGAL urine - ARCHITECT) | £23,110 | Dominated | 6.07534 | Dominated | Dominated | Dominated | 0.0% | 0.0% |
| Test 1 (NephroCheck) | £23,145 | Dominated | 6.07534 | Dominated | Dominated | Dominated | 0.0% | 0.0% |
| **Scenario 3: Assume NGAL can also avert AKI** | | | | | | | | |
| Test 3 (NGAL urine - BioPorto) | £22,887 | -- | 6.07332 | -- | -- | Dominant | 43.5% | 54.6% |
| Test 2 (NGAL plasma - BioPorto) | £22,900 | £14 | 6.07332 | 0.00001 | £2,694,918 | Dominant | 11.1% | 47.6% |
| Standard care (Scr) | £22,901 | Dominated | 6.07296 | Dominated | Dominated | -- | 45.1% | -- |
| Test 4 (NGAL urine - ARCHITECT) | £22,912 | Dominated | 6.07328 | Dominated | Dominated | £32,131 | 0.1% | 41.4% |
| Test 1 (NephroCheck) | £22,938 | Dominated | 6.07332 | Dominated | Dominated | £101,456 | 0.2% | 31.9% |
| **Scenario 4: As per Scenario 1 (full associative effect) but apply a daily excess AKI costs to patients in hospital/ICU** | | | | | | | | |
| Standard care (Scr) | £23,729 | -- | 6.08549 | -- | -- | -- | 0.7% | -- |
| Test 1 (NephroCheck) | £23,730 | £1 | 6.11261 | 0.02712 | £29 | £29 | 98.8% | 99.1% |
| Test 3 (NGAL urine - BioPorto) | £23,815 | Dominated | 6.09063 | Dominated | Dominated | £16,615 | 0.5% | 54.0% |
| Test 4 (NGAL urine - ARCHITECT) | £23,830 | Dominated | 6.09020 | Dominated | Dominated | £21,436 | 0.0% | 45.1% |
| Test 2 (NGAL plasma - BioPorto) | £23,831 | Dominated | 6.09079 | Dominated | Dominated | £19,153 | 0.0% | 49.9% |
| **Scenario 5: Exclude RRT cost** | | | | | | | | |
| Standard care (Scr) | £22,779 | -- | 6.07846 | -- | -- | -- | 68.1% | -- |
| Test 1 (NephroCheck) | £22,823 | £43 | 6.07882 | 0.00036 | £119,317 | £119,317 | 27.7% | 29.6% |
| Test 3 (NGAL urine - BioPorto) | £22,850 | Dominated | 6.07859 | Dominated | Dominated | £533,230 | 3.8% | 9.0% |
| Test 2 (NGAL plasma - BioPorto) | £22,865 | Dominated | 6.07859 | Dominated | Dominated | £633,002 | 0.4% | 6.8% |
| Test 4 (NGAL urine - ARCHITECT) | £22,867 | Dominated | 6.07858 | Dominated | Dominated | £730,093 | 0.0% | 5.6% |
| **Scenario 6: Apply the differential long-term follow-up costs and mortality according to whether patient entered ICU or not** | | | | | | | | |
| Test 1 (NephroCheck) | £30,438 | -- | 6.55843 | -- | -- | Dominant | 97.2% | 97.2% |
| Standard care (Scr) | £30,712 | Dominated | 6.55697 | Dominated | Dominated | -- | 2.8% | -- |
| Test 3 (NGAL urine - BioPorto) | £30,776 | Dominated | 6.55733 | Dominated | Dominated | £181,324 | 0.0% | 15.4% |
| Test 2 (NGAL plasma - BioPorto) | £30,790 | Dominated | 6.55733 | Dominated | Dominated | £217,350 | 0.0% | 11.8% |
| Test 4 (NGAL urine - ARCHITECT) | £30,793 | Dominated | 6.55730 | Dominated | Dominated | £249,264 | 0.0% | 9.3% |
| **Scenario 7: Apply an excess CKD risk for those who experienced an AKI event over the full lifetime horizon** | | | | | | | | |
| Test 1 (NephroCheck) | £23,172 | -- | 6.07060 | -- | -- | Dominant | 55.5% | 57.7% |
| Standard care (Scr) | £23,174 | Dominated | 6.06893 | Dominated | Dominated | -- | 39.9% | -- |
| Test 3 (NGAL urine - BioPorto) | £23,231 | Dominated | 6.06947 | Dominated | Dominated | £106,920 | 3.6% | 21.2% |
| Test 2 (NGAL plasma - BioPorto) | £23,246 | Dominated | 6.06948 | Dominated | Dominated | £132,282 | 1.0% | 16.6% |
| Test 4 (NGAL urine - ARCHITECT) | £23,250 | Dominated | 6.06942 | Dominated | Dominated | £154,900 | 0.0% | 12.7% |
| **Scenario 8: 0% discount rate applied to both costs and QALYs** | | | | | | | | |
| Standard care (Scr) | £27,689 | -- | 8.20138 | -- | -- | -- | 60.5% | -- |
| Test 1 (NephroCheck) | £27,717 | £28 | 8.20191 | 0.00053 | £52,565 | £52,565 | 34.1% | 36.6% |
| Test 3 (NGAL urine - BioPorto) | £27,757 | Dominated | 8.20157 | Dominated | Dominated | £371,108 | 4.9% | 12.7% |
| Test 2 (NGAL plasma - BioPorto) | £27,771 | Dominated | 8.20157 | Dominated | Dominated | £439,959 | 0.4% | 9.5% |
| Test 4 (NGAL urine - ARCHITECT) | £27,774 | Dominated | 8.20155 | Dominated | Dominated | £500,966 | 0.1% | 7.0% |
| **Scenario 9: 6% discount rate applied to both costs and QALYs** | | | | | | | | |
| Standard care (Scr) | £21,153 | -- | 5.11027 | -- | -- | -- | 67.1% | -- |
| Test 1 (NephroCheck) | £21,192 | £40 | 5.11055 | 0.00028 | £140,771 | £140,771 | 27.4% | 30.7% |
| Test 3 (NGAL urine - BioPorto) | £21,221 | Dominated | 5.11037 | Dominated | Dominated | £686,941 | 4.7% | 10.8% |
| Test 2 (NGAL plasma - BioPorto) | £21,235 | Dominated | 5.11038 | Dominated | Dominated | £808,828 | 0.8% | 8.0% |
| Test 4 (NGAL urine - ARCHITECT) | £21,238 | Dominated | 5.11036 | Dominated | Dominated | £937,507 | 0.0% | 6.3% |
| **Scenario 10: Apply alternative source for AKI prevalence (average prevalence 0.2332 across systematic review studies)** | | | | | | | | |
| Test 1 (NephroCheck) | £23,014 | -- | 5.85682 | -- | -- | Dominant | 63.1% | 67.0% |
| Standard care (Scr) | £23,122 | Dominated | 5.85589 | Dominated | Dominated | -- | 28.4% | -- |
| Test 3 (NGAL urine - BioPorto) | £23,171 | Dominated | 5.85623 | Dominated | Dominated | £142,617 | 6.7% | 33.2% |
| Test 2 (NGAL plasma - BioPorto) | £23,183 | Dominated | 5.85624 | Dominated | Dominated | £174,191 | 1.8% | 30.1% |
| Test 4 (NGAL urine - ARCHITECT) | £23,188 | Dominated | 5.85620 | Dominated | Dominated | £211,691 | 0.0% | 26.1% |
| **Scenario 11: Increase the number of times test is conducted to 2** | | | | | | | | |
| Standard care (Scr) | £22,746 | -- | 6.07904 | -- | -- | -- | 88.8% | -- |
| Test 3 (NGAL urine - BioPorto) | £22,873 | Ext Dom | 6.07916 | Ext Dom | Ext Dom | £1,053,861 | 1.9% | 2.6% |
| Test 1 (NephroCheck) | £22,875 | £129 | 6.07939 | 0.00035 | £369,737 | £369,737 | 9.0% | 9.4% |
| Test 2 (NGAL plasma - BioPorto) | £22,888 | Dominated | 6.07916 | Dominated | Dominated | £1,167,690 | 0.3% | 1.5% |
| Test 4 (NGAL urine - ARCHITECT) | £22,898 | Dominated | 6.07915 | Dominated | Dominated | £1,370,281 | 0.0% | 0.7% |
| **Scenario 12: Apply an additional risk of mortality to those with a false positive test (RR=1.5)** | | | | | | | | |
| Test 1 (NephroCheck) | £22,533 | -- | 5.93052 | -- | -- | £3,062 | 0% | 0% |
| Test 2 (NGAL plasma - BioPorto) | £22,632 | £99 | 5.94584 | 0.01532 | £6,478 | £2,644 | 0% | 0% |
| Test 4 (NGAL urine - ARCHITECT) | £22,715 | £83 | 5.97024 | 0.02440 | £3,389 | £2,464 | 0% | 0% |
| Test 3 (NGAL urine - BioPorto) | £22,809 | £94 | 6.00383 | 0.03360 | £2,801 | £2,297 | 0% | 0% |
| Standard care (Scr) | £22,963 | £155 | 6.07124 | 0.06740 | £2,297 | -- | 100% | -- |
| **Scenario 13: Exclude capital and training costs in test costs** | | | | | | | | |
| Standard care (Scr) | £22,987 | -- | 6.08128 | -- | -- | -- | 65.1% | -- |
| Test 1 (NephroCheck) | £23,025 | £39 | 6.08162 | 0.00035 | £111,620 | £111,620 | 29.4% | 32.2% |
| Test 3 (NGAL urine - BioPorto) | £23,051 | Dominated | 6.08139 | Dominated | Dominated | £546,618 | 4.5% | 12.6% |
| Test 2 (NGAL plasma - BioPorto) | £23,066 | Dominated | 6.08140 | Dominated | Dominated | £663,328 | 1.0% | 9.3% |
| Test 4 (NGAL urine - ARCHITECT) | £23,069 | Dominated | 6.08138 | Dominated | Dominated | £766,927 | 0.0% | 6.1% |
| **Scenario 14: Apply alternative ICU utility value (average of -0.402 and 0.44)** | | | | | | | | |
| Standard care (Scr) | £23,234 | -- | 6.07749 | -- | -- | -- | 67.2% | -- |
| Test 1 (NephroCheck) | £23,274 | £41 | 6.07783 | 0.00034 | £120,580 | £120,580 | 28.0% | 29.9% |
| Test 3 (NGAL urine - BioPorto) | £23,302 | Dominated | 6.07761 | Dominated | Dominated | £586,840 | 4.4% | 11.0% |
| Test 2 (NGAL plasma - BioPorto) | £23,317 | Dominated | 6.07761 | Dominated | Dominated | £696,184 | 0.4% | 8.1% |
| Test 4 (NGAL urine - ARCHITECT) | £23,319 | Dominated | 6.07760 | Dominated | Dominated | £796,431 | 0.0% | 6.2% |
| **Scenario 15: Alternative outpatient utility source in the long term (apply general population norms)** | | | | | | | | |
| Standard care (Scr) | £22,867 | -- | 7.05869 | -- | -- | -- | 63.5% | -- |
| Test 1 (NephroCheck) | £22,904 | £36 | 7.05928 | 0.00059 | £61,809 | £61,809 | 32.0% | 34.1% |
| Test 3 (NGAL urine - BioPorto) | £22,938 | Dominated | 7.05889 | Dominated | Dominated | £360,613 | 4.3% | 9.9% |
| Test 2 (NGAL plasma - BioPorto) | £22,954 | Dominated | 7.05889 | Dominated | Dominated | £431,098 | 0.2% | 7.8% |
| Test 4 (NGAL urine - ARCHITECT) | £22,955 | Dominated | 7.05887 | Dominated | Dominated | £483,707 | 0.0% | 5.7% |

Ext Dom: Extendedly dominated; P(C/E): probability that a test is cost-effective at a threshold value of willingness to pay for a QALY of £20,000.

## Supplementary Table 5 Sensitivity and specificity data obtained from the systematic review for the subgroup analysis

| **Test** | **Parameter** | **Mean value**  **(95% CI)** | **Mean (logit scale)** | **Standard error (logit scale)** | **Correlation for MVN distribution (logit scale)** |
| --- | --- | --- | --- | --- | --- |
| **Critical care group ^A^** | | | | | |
| NephroCheck | Sensitivity | 0.83  (0.72 to 0.91) | 1.615 | 0.336 | -1.000 |
|  | Specificity | 0.51  (0.48 to 0.54) | 0.040 | 0.064 |  |
| NGAL urine (BioPorto) | Sensitivity | 0.72  (0.61 to 0.80) | 0.926 | 0.247 | +0.905 |
|  | Specificity | 0.87  (0.66 to 0.96) | 1.876 | 0.617 |  |
| NGAL urine Abbot ARCHITECT | Sensitivity | 0.70  (0.63 to 0.76) | 0.855 | 0.165 | +1.000 |
|  | Specificity | 0.72  (0.63 to 0.80) | 0.958 | 0.226 |  |
| NGAL plasma (BioPorto) | Sensitivity | 0.76  (0.56 to 0.89) | 1.156 | 0.462 | -1.000 |
|  | Specificity | 0.67  (0.40 to 0.86) | 0.686 | 0.566 |  |
| **Cardiac surgery group ^A^** | | | | | |
| NephroCheck ^B^ | Sensitivity | 0.31  (0.09 to 0.61) | -0.800 | 0.704 | -0.824 ^F^ |
|  | Specificity | 0.78  (0.74 to 0.82) | 1.266 | 0.120 |  |
| NGAL urine  (BioPorto) ^C^ | Sensitivity | 0.78  (0.72 to 0.84) | 1.266 | 0.182 | +0.526 ^F^ |
|  | Specificity | 0.48  (0.42 to 0.54) | -0.080 | 0.123 |  |
| NGAL urine  (ARCHITECT) ^D^ | Sensitivity | 0.46  (0.33 to 0.59) | -0.160 | 0.274 | -0.517 ^F^ |
|  | Specificity | 0.81  (0.79 to 0.83) | 1.450 | 0.067 |  |
| NGAL plasma  (BioPorto) ^E^ | Sensitivity | 0.62  (0.49 to 0.74) | 0.490 | 0.277 | -1.000 ^F^ |
|  | Specificity | 0.78  (0.75 to 0.81) | 1.266 | 0.090 |  |

^A^ For the subgroup analyses, the same model parameter values were used as in the base case analysis, with the exception of the diagnostic accuracy data presented here. Although one of these subgroups is described as being in critical care, the population described in the diagnostic accuracy studies is more in line with a severely ill patient group not yet in an ICU in a UK setting. The diagnostic accuracy data for the cardiac group was more limited. Single studies where used were possible from the diagnostic accuracy review. Where data was not available, we applied pooled estimates from Hall et al. (24)

^B^ Based on test accuracy data from Cummings et al. 2019 (28)

^C^ Based on test accuracy data from Yang et al. 2017 (29)

^D^ Based on test accuracy data from Parikh et al. 2017 (30)

^E^ Based on test accuracy data from Hall et al. 2018 (24)

^F^ Due to the lack of data for the cardiac surgery subgroup and the absence of a meta-analysis, all correlations were assumed to be equal to the base case values.

## Supplementary Table 6 Results of the subgroup analyses

| **Scenario** | **Cost** | **Incremental Cost** | **QALY** | **Incremental QALY** | **ICER (incremental)** | **ICER vs. standard care** | **p (C/E) @ 20k** | **p (C/E) @ 20k vs. standard care** |
| --- | --- | --- | --- | --- | --- | --- | --- | --- |
| **Critical care subgroup** | | | | | | | | |
| Standard care  (Scr) | £22,904 |  | 6.07716 |  |  |  | 65.0% | -- |
| Test 1 (NephroCheck) | £22,937 | £32 | 6.07755 | 0.00039 | £82,079 | £82,079 | 31.4% | 32.8% |
| Test 3 (NGAL urine - BioPorto) | £22,971 | Dominated | 6.07728 | Dominated | Dominated | £555,173 | 3.0% | 11.1% |
| Test 2 (NGAL plasma - BioPorto) | £22,991 | Dominated | 6.07729 | Dominated | Dominated | £676,218 | 0.5% | 8.3% |
| Test 4 (NGAL urine - ARCHITECT) | £22,991 | Dominated | 6.07728 | Dominated | Dominated | £732,572 | 0.1% | 7.9% |
| **Cardiac care subgroup** | | | | | | | | |
| Standard care  (Scr) | £22,983 |  | 6.07043 |  |  |  | 85.6% |  |
| Test 2 (NGAL plasma - BioPorto) | £23,055 | Ext Dom | 6.07054 | Ext Dom | Ext Dom | £679,042 | 3.8% | 8.4% |
| Test 1 (NephroCheck) | £23,057 | £74 | 6.07059 | 0.00016 | £465,544 | £465,544 | 6.5% | 8.1% |
| Test 4 (NGAL urine - ARCHITECT) | £23,062 | Dominated | 6.07051 | Dominated | Dominated | £996,121 | 0.1% | 4.0% |
| Test 3 (NGAL urine - BioPorto) | £23,082 | Dominated | 6.07056 | Dominated | Dominated | £737,663 | 4.0% | 7.5% |

Dominated: more costly and less effective; Ext Dom: Extendedly dominated; P(C/E): probability that a test is cost-effective at a threshold value of willingness to pay for a QALY of £20,000.

**References**

(1) Sawhney S, Marks A, Fluck N, Levin A, Prescott G, Black C. Intermediate and long-term outcomes of survivors of acute kidney injury episodes: a large population-based cohort study. American Journal of Kidney Diseases 2017;69(1):18-28.

(2) Sawhney S, Robinson HA, van der Veer, S. N., Hounkpatin HO, Scale TM, Chess JA, et al. Acute kidney injury in the UK: a replication cohort study of the variation across three regional populations. BMJ Open 2018 Jun 30;8(6):e019435-019435.

(3) Truche A, Ragey SP, Souweine B, Bailly S, Zafrani L, Bouadma L, et al. ICU survival and need of renal replacement therapy with respect to AKI duration in critically ill patients. Annals of intensive care 2018;8(1):127.

(4) Bastin AJ, Ostermann M, Slack AJ, Diller G, Finney SJ, Evans TW. Acute kidney injury after cardiac surgery according to risk/injury/failure/loss/end-stage, acute kidney injury network, and kidney disease: improving global outcomes classifications. J Crit Care 2013;28(4):389-396.

(5) Meersch M, Schmidt C, Hoffmeier A, Van Aken H, Wempe C, Gerss J, et al. Prevention of cardiac surgery-associated AKI by implementing the KDIGO guidelines in high risk patients identified by biomarkers: the PrevAKI randomized controlled trial. Intensive Care Med 2017;43(11):1551-1561.

(6) See EJ, Jayasinghe K, Glassford N, Bailey M, Johnson DW, Polkinghorne KR, et al. Long-term risk of adverse outcomes after acute kidney injury: a systematic review and meta-analysis of cohort studies using consensus definitions of exposure. Kidney Int 2019;95(1):160-172.

(7) Rimes-Stigare C, Frumento P, Bottai M, Mårtensson J, Martling C, Walther SM, et al. Evolution of chronic renal impairment and long-term mortality after de novo acute kidney injury in the critically ill; a Swedish multi-centre cohort study. Critical care 2015;19(1):221.

(8) Kent S, Schlackow I, Lozano-Kühne J, Reith C, Emberson J, Haynes R, et al. What is the impact of chronic kidney disease stage and cardiovascular disease on the annual cost of hospital care in moderate-to-severe kidney disease? BMC nephrology 2015;16(1):65.

(9) Lone NI, Gillies MA, Haddow C, Dobbie R, Rowan KM, Wild SH, et al. Five-year mortality and hospital costs associated with surviving intensive care. American Journal of Respiratory and Critical Care Medicine 2016;194(2):198-208.

(10) Office for National Statistics. National life tables: UK [online]. Newport, Wales: Office of National Statistics. 2019. Available from: https://www.ons.gov.uk/peoplepopulationandcommunity/birthsdeathsandmarriages/lifeexpectancies/datasets/nationallifetablesunitedkingdomreferencetables. Accessed 31 October 2019.

(11) UK Renal Registry. UK Renal Registry 21st Annual Report - data to 31/12/2017 [online]. Bristol, UK: UK Renal Registry. Available from: https://www.renalreg.org/publications-reports/. Accessed 24 October 2019.

(12) Joint Formulary Committee. 2019. British National Formulary [Online] London: BMJ Group and Pharmaceutical Press. Available from: https://bnf.nice.org.uk/medicinal-forms/sodium-chloride.html#PHP76736. Accessed 25 October 2019.

(13) Personal Social Services Research Unit. 2018. *Unit Costs of Health and Social Care* [Online] Canterbury, Kent: University of Kent. Available from: <https://www.pssru.ac.uk/project-pages/unit-costs/>. Accessed 9 September 2019.

(14) NHS Improvement. 2017. Reference costs [Online] London: NHS Improvement. Available from: https://improvement.nhs.uk/resources/reference-costs/. Accessed 9 September 2019.

(15) Sarwar S, Shafi MI. National Confidential Enquiry into Patient outcome and death. Obstetrics, Gynaecology & Reproductive Medicine 2007;17(9):278-279.

(16) Hernández RA, Jenkinson D, Vale L, Cuthbertson BH. Economic evaluation of nurse-led intensive care follow-up programmes compared with standard care: the PRaCTICaL trial. The European Journal of Health Economics 2014;15(3):243-252.

(17) National Institute for Health and Care Excellence. 2015. NICE. Kidney Transplantation (Adults) – Immunosuppressive Therapy (Review Of TA 85) [ID456] [Online] London/Manchester. Available from: [www.nice.org.uk/guidance/GID-TAG348/documents/html-content](http://www.nice.org.uk/guidance/GID-TAG348/documents/html-content). Accessed November 2016.

(18) Kind P, Hardman G, Macran S. UK population norms for EQ-5D. York: Centre for Health Economics. 1999. Available from: https://www.york.ac.uk/che/pdf/DP172.pdf. Accessed 26 October 2019.

(19) Wyld M, Morton RL, Hayen A, Howard K, Webster AC. A systematic review and meta-analysis of utility-based quality of life in chronic kidney disease treatments. PLoS Med 2012;9(9):e1001307.

(20) Cuthbertson BH, Roughton S, Jenkinson D, MacLennan G, Vale L. Quality of life in the five years after intensive care: a cohort study. Critical care 2010;14(1):R6.

(21) Nguyen NTQ, Cockwell P, Maxwell AP, Griffin M, O'Brien T, O'Neill C. Chronic kidney disease, health-related quality of life and their associated economic burden among a nationally representative sample of community dwelling adults in England. PLoS One 2018 Nov 26;13(11):e0207960.

(22) Liem YS, Bosch JL, Hunink MM. Preference-based quality of life of patients on renal replacement therapy: a systematic review and meta-analysis. Value in Health 2008;11(4):733-741.

(23) Ara R, Brazier JE. Populating an economic model with health state utility values: moving toward better practice. Value in Health 2010;13(5):509-518.

(24) Hall PS, Mitchell ED, Smith AF, Cairns DA, Messenger M, Hutchinson M, et al. The future for diagnostic tests of acute kidney injury in critical care: evidence synthesis, care pathway analysis and research prioritisation. Health Technol Assess 2018(22(32)):1-274.

(25) UK Renal Registry. 2018. UK Renal Registry 20th Annual Report - data to 31/12/2016 [online]. Bristol, UK: UK Renal Registry. Available from: https://www.renalreg.org/publications-reports/. Accessed 24 October 2019.

(26) Wilson FP, Shashaty M, Testani J, Aqeel I, Borovskiy Y, Ellenberg SS, et al. Automated, electronic alerts for acute kidney injury: a single-blind, parallel-group, randomised controlled trial. The Lancet 2015;385(9981):1966-1974.

(27) National Institute for Health and Care Excellence. Tests to help assess risk of acute kidney injury for people being considered for critical care admission (ARCHITECT and Alinity i Urine NGAL assays, BioPorto NGAL test and NephroCheck test) Diagnostics guidance [DG39]. 2020. Available at: https://www.nice.org.uk/guidance/dg39/history. Accessed 3 November 2020.

(28) Cummings JJ, Shaw AD, Shi J, Lopez MG, O'Neal JB, Billings IV FT. Intraoperative prediction of cardiac surgery–associated acute kidney injury using urinary biomarkers of cell cycle arrest. . The Journal of thoracic and cardiovascular surgery 2019 Apr;157(4):1545-53.

(29) Yang X, Chen C, Teng S, Fu X, Zha Y, Liu H, Wang L, Tian J, Zhang X, Liu Y, Nie J. Urinary matrix metalloproteinase-7 predicts severe AKI and poor outcomes after cardiac surgery. Journal of the American Society of Nephrology 2017 Nov;28(11):3373-82.

(30) Parikh A, Rizzo JA, Canetta P, Forster C, Sise M, Maarouf O, et al. Does NGAL reduce costs? A cost analysis of urine NGAL (uNGAL) & serum creatinine (sCr) for acute kidney injury (AKI) diagnosis. PLoS One 2017 May 19;12(5):e0178091.
